# Vascular Endothelial Growth Factor in Children with Thalassemia Major

**DOI:** 10.4084/MJHID.2013.044

**Published:** 2013-06-05

**Authors:** Sameh S. Fahmey, Hassan F. Naguib, Sanna S. Abdelshafy, Rasha E. Alashry

**Affiliations:** 1 Pediatrics Department, Beni Suef University.; 2 Clinical Pathology Department, Beni Suef University.

## Abstract

**Background:**

The β-Thalassemia syndromes are the most common hereditary chronic hemolytic anemia due to impaired globin chain synthesis. Vascular endothelial growth factor (VEGF) plays several roles in angiogenesis which is a crucial process in the pathogenesis of several inflammatory, autoimmune and malignant diseases. Endothelial damage and inflammation make a significant contribution to the pathophysiology of β-thalassemia.

**Purpose:**

: The aim of the study was to assess serum VEGF level in children with beta-thalassemia major as a marker of angiogenesis.

**Methods:**

A total of 50 children entered the study, 40 patients with thalassemia major and 10 healthy controls. We used enzyme-linked immunosorbent assay for quantitative evaluation of VEGF.

**Results:**

VEGF level was significantly higher in patients with β-thalassemia major than healthy controls (p=0.001). VEGF level was also higher in splenectomised thalassemic patients than non splenectomised ones (p=0.001). There were a positive correlation between VEGF and chelation starting age (p=0.008), and a negative correlation between VEGF and frequency of blood transfusion (p=0.002).

**Conclusion:**

Thalassemia patients, especially splenectomized, have elevated serum levels of VEGF. Early chelation and regular blood transfusion help to decrease serum VEGF and the risk of angiogenesis.

## Introduction

Beta-thalassemia major is an autosomal recessive hereditary anemia, which is incurable, caused by defective synthesis of hemoglobin, ineffective erythropoiesis, and rapid erythrocyte breakdown.^1^

Beta-thalassemia major patients frequently end up with iron overload because of hemolysis and repeated blood transfusion. Treatment with iron chelating therapy in patients with beta-thalassemia is considered the standard care, leading to improvement of morbidity and increased rate of survival.^2^

Arterial and venous thromboembolic episodes in beta-thalassemia major patients have been reported. Endothelial cell activation and impaired flow-mediated dilation in the brachial arteries of beta-thalassemic patients, as shown in previous in vivo studies, implicate endothelial dysfunction in the pathogenesis of vascular complications. Endothelial dysfunction generally leads to vascular remodeling and potential changes in mechanical properties.^3^

Endothelial cell proliferation plays a role in vascular injury repair and blood vessels formations. It is affected by plasma derived and blood cell derived component.^4^

Angiogenesis, or the growth of new blood vessels, is important for wound healing and for restoring blood flow to tissues after injury or insult. In normal physiology, inhibitors and angiogenic growth factors, such as vascular endothelial growth factor (VEGF), regulate angiogenesis. When regulation fails, blood vessels are formed excessively or insufficiently.^5^

Tissue hypoxia is a major stimulus for the up-regulation of VEGF and anemic patients have elevated levels of VEGF. This suggests that anemia might impact on the progression of angeiogenesis in malignant and benign diseases.^6^

The aim of the study was to assess serum VEGF level in children with beta-thalassemia major as a marker of angiogenesis.

## Material and Methods

The population of the study consisted of 40 patients with beta-thalassemia major and 10 healthy, age and sex matched controls. Patients with beta-thalassemia major were recruited from the hematology clinic of Beni Suef University Hospital in the period from May through October 2012. The patients were diagnosed as beta-Thalassemia major based on clinical and hematological characteristics (CBC and hemoglobin electrophoresis). None of the patients had received a blood transfusion within the 3 weeks before the study. Subjects with other hemoglobinopathy, malignancy or other causes of anemia were excluded from the study. Ethical clearance was obtained from the ethical committee of the hospital. Parents of all participating children gave written consent to their child’s participation in the study. All cases were subjected to detailed history including age, sex, duration of illness, frequency of blood transfusion, type of chelation therapy and history of splenectomy. Clinical examination included anthropometrics measurements, vital signs and presence of any complications. Laboratory investigations included complete blood picture, serum ferritin and serum VEGF.

### Sample collection and VEGF assay

Blood samples were drawn from patients by vacutainer tubes. The samples were centrifuged for 10 minutes then sera were separated and stored at −70°C. Thereafter, VEGF levels were measured by enzyme-linked immunosorbent assay using the Orgenium Laboratories’ Human VEGF ELISA kit in accordance with the manufacturer’s instructions. The detection limit of the VEGF assay was 9 pg/ml, the intra-assay precision was ≤ 6 % and the inter-assay precision was ≤10%.

Serum VEGF corrected for platelet count was calculated as serum VEGF (pg/ml) / platelet count (10^3^/μL) to exclude the effect of the platelet count.

### Statistical Analysis

Statistical analysis was performed using Statistical Package for Social Science (SPSS) software version 17. Quantitative variables were expressed as mean and standard deviation. Qualitative variables were expressed as count and percentage. Cross tabulation test was used for comparison between percentage values. Student t- test was used for comparison between means of two groups. Mann-Whitney U test was used for two independent samples. The Pearson correlation coefficient test used to test the significant correlations between the quantitative parameters within each group. A P value less than 0.05 was considered significant.

## Results

The demographic and laboratory data of patients are shown in Table 1. A total of 40 patients were enrolled in the study, 15(37.5 %) of them were female and 25 (62.5%) were male. Their age ranged between 1.9 and 14 years. Their mean weight was 24.4±3.8 kg and mean height was 119.5±18.7.

The control group consisted of 10 healthy children (5 males and 5 females).Their mean values were as follow: age, 7.8±1.7 years; weight, 28±4.3 kgs; leukocyte (WBC) counts, 9.8±2.8 10^3^/μL; hemoglobin, 14.3±3.7 g/dl; platelets, 205±95 10^3^/μL and ferritin, 115.2±60.0 ng/ml.

Serum VEGF levels were 1241.5±632.9 and 438.8±191.03 in patients and controls respectively (p<0.001) (Table 2).

16 patients had splenectomy (40%) and hepatomegaly was found in 18 patients (45%). Platelet counts were 592.1±279.8 (10^3^/μL) and 299.2±148.9 (10^3^/μL) in patients with and without splenectomy, respectively (p=0.001). Table 3 shows comparison between serum VEGF in patients with and without splenectomy. A significant correlation was observed between VEGF level and platelets count (Table 4).

As regards the chelating drugs, desferrioxamine (n = 4), deferasirox (n = 7), deferiprone (n = 25) and deferiprone plus desferrioxamine (n = 4) were used for chelation. However, serum VEGF was not affected by the type of chelating drug used (p>0.05) but we found a positive correlation between serum VEGF and chelation starting age (p=0.008).

**Figure 1:**
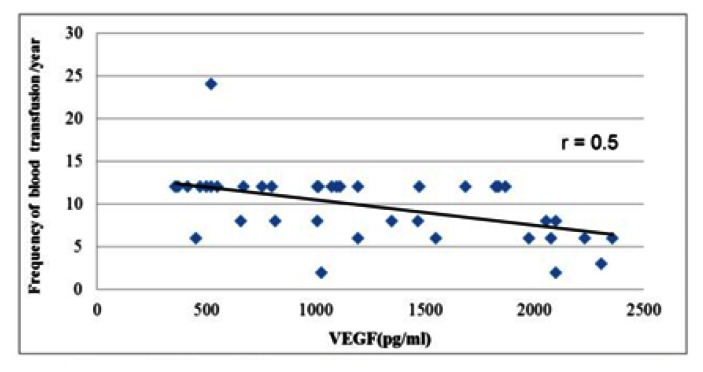
Correlation between frequency of blood transfusion and serum vascular endothelial growth factor(r=0.5).

We did not find a significant correlation between VEGF level and the disease duration (p>0.05), but there was a negative correlation between VEGF level and the frequency of blood transfusion (p=0.002) (Figure 1).

## Discussion

The higher standards of care in β-thalassemia have led to significant increase in the life expectancy in the severely affected patients. Enhanced years of survival have led to the unmasking of management related complications, which were infrequently encountered.^7^

Inflammation is known to have an important role in the pathogenesis of thalassemia. A chronic inflammatory state is present in these patients.^8^

Endothelial activation is also believed to play an important role in the pathophysiology of thalassemia, through inflammation and thrombosis.^9^

VEGF is mitogenic for endothelial cells and promotes vascular leakage. Besides its activity on endothelial cell proliferation, VEGF has synergistic activity with tumor necrosis factor (TNF) in inducing procoagulant activity of endothelial cells, promotes migration of monocytes across endothelial cells monolayers, and causes Von Willebrand factor release. Thus, VEGF affects endothelial functions related both to angiogenesis and to inflammation and thrombosis.^10^ Patients with thalassemia, whether splenectomized or not, are prone to the development of pulmonary thrombosis and inflammation.^11^ However, none of our thalassemic patients had thrombotic events.

Angiogenesis has been investigated in sickle cell disease (SCD) but limited studies had discussed angiogenesis in patients with β-thalassemia major. In this study, the serum level of vascular endothelial growth factor (VEGF) was found significantly higher compared to healthy individuals. This finding is in agreement with Voskaridou et al^12^ who reported that patients with thalassemia major had increased levels of all studied angiogenic cytokines (such as VEGF, basic fibroblast growth factor, angiogenin, angiopoietin) compared with healthy controls. Also, Butthep et al^13^ reported that thalassemia patients are characterized by increased levels of VEGF and TNF.

As regards SCD, Mohan et al^14^ reported elevated VEGF plasma levels in clinically asymptomatic SCD patients.

Elevated serum levels of VEGF in thalassemia patient can be explained by tissue hypoxia which is the main stimulus for the up-regulation of VEGF.^6^

In our study, there was a significant difference in VEGF level between splenectomized and non splenectomized patients (p=0.001), being higher in patients underwent splenectomy. Similar observations were reported by Shitrit et al^4^ who demonstrated high level of VEGF in splenectomy group. Our current explanation is that splenectomized patients have a higher platelets count which act as a reservoir for VEGF. The impact of platelets on serum levels of VEGF has been previously described^15^,^16^ and was supported in this study. We found a strong correlation between VEGF and platelets count (p=0.001).

Serum VEGF corrected by platelets (to exclude the effect of the platelet count) was significantly higher in splenectomized compared to non-splenectomized patients (p=0.02).So, higher serum level of VEGF in splenectomized patients may also be explained by the disease severity as defined by the need for splenectomy.

Ferritin, through a direct interaction with both HK (high molecular weight kininogen) and HKa(two-chain high molecular weight kininogen), is a newly defined angiogenic regulator. Through binding to the anti-angiogenic domain of HKa, ferritin antagonizes HKa’s effects, leading to increased blood vessel growth..^17^ However, we did not find a significant correlation between VEGF and ferritin. This might be due to the effect of chelation therapies.

We failed to show a significant correlation between hemoglobin levels and VEGF, which might be due to the fact that those patients were on regular blood transfusion.

According to our results, there is a strong inverse correlation between VEGF and blood transfusion frequency (p=0.002).Therefore, regular blood transfusion helps to decrease angiogenesis. Moreover, we found a strong positive correlation between VEGF and chelation starting age (p=0.008).So, the early use of chelation therapy will help to decrease angiogenesis. However, we did not find a significant correlation between VEGF and the chelating drugs used. These data go in concordance with previous study by Olgar et al.^18^

In our study, there was no significant correlation between serum level of VEGF and the duration of disease (p=0.072).However, no available studies have specifically examined the relation between VEGF and duration of disease in thalassemia major.

## Conclusions

thalassemia patients, especially splenectomized, have elevated serum levels of VEGF. Early chelation and regular blood transfusion help to decrease serum VEGF and the risk of angiogenesis.

## Figures and Tables

**Table 1:** Demographic and laboratory data of patients (n=40)

Parameter	Value(mean±SD)
Age(years)	8.5±1.1
Weight(kg)	24.4±3.8
Height(cm)	119.5±18.7
Age at diagnosis(years)	1.6±0.7
Age at chelation onset (years)	3.1±4
Hemoglobin (g/dl)	7.7±1.4
Hematocrit (%)	23±5.3
WBC(10^3^/μL)	14.5±3.7
Platelets(10^3^/μL)	416.3±253.6
Serum ferritin(ng/ml)	1093.7±868.6
Serum VEGF(pg/ml)	1241.5±632.9

**Table 2:** comparison of serum vascular endothelial growth factor (VEGF) levels in patients and controls.

	Patients (mean±SD)	Controls (mean±SD)	*p-*value
VEGF(pg/ml)	1241.5±632.9	438.8±191.03	< 0.001
VEGF corrected by platelets (pg/10^3^)	3.47 ± 1.94	2.26 ± 0.69	0.003

**Table 3:** Comparison between serum vascular endothelial growth factor (VEGF) in patients with and without splenectomy.

	With splenectomy (mean±SD)	Without splenectomy (mean±SD)	*p*-value
Serum VEGF(pg/ml)	1680.4±531.7	948±521.03	0.001
VEGF corrected by platelets(pg/10^3^)	4.52±2.53	2.86±1.07	0.02

**Table 4:** correlation of laboratory findings with serum vascular endothelial growth factor.

	Pearson correlation (r)	*p*-value
Hemoglobin	0.06	0.703
Hematocrit	−0.14	0.392
White blood cells	0.22	0.172
Platelets	0.60	0.001
Serum ferritin level	0.10	0.557
